# Executive functions as mediators of early educational disparities by SES, gender and birth month

**DOI:** 10.1038/s41539-026-00422-w

**Published:** 2026-04-16

**Authors:** Margot Rémeau, Philippe Schmitt, Grégoire Borst

**Affiliations:** 1 Université Paris Cité, LaPsyDÉ, CNRS, Paris, France; 2 Direction de l’évaluation, de la prospective et de la performance (DEPP), ministère de l’Éducation nationale, Paris, France; 3 Institut Universitaire de France, Paris, France

**Keywords:** Education, Psychology

## Abstract

Educational inequalities emerge early, yet the combined influence of socioeconomic status (SES), gender, and birth month remains understudied in preschool. While SES-related disparities have been linked to executive functions (EFs)—cognitive processes supporting goal-directed behavior—their role in gender- and birth-month-related inequalities is unknown. In a sample of 2,618 preschoolers (mean age = 42.61 months, SD = 3.41; girls/boys = 1254/1364), SES, gender, and birth month each independently predicted pre-academic abilities. EFs significantly mediated these associations, explaining 23% of SES-related disparities, 39% of gender-related disparities, and 51% of birth-month-related disparities. These findings provide novel evidence that early educational inequalities may partly originate from EF differences, underscoring their central role in shaping school readiness.

## Introduction

Educational systems around the world are characterized by persistent inequalities which represents a challenge for democratic societies seeking to promote equal opportunities for all^1^. Given the well-established link between academic success and lifelong development, addressing these disparities is both a societal priority and an important area of inquiry in educational research^2,3^. According to the Organization for Economic Co-operation and Development (OECD) variability in individuals’ life outcomes is influenced by a complex interplay of factors operating at different levels^1^. These factors include individual characteristics (e.g., gender, socio-economic status, cognitive and socio-emotional skills), the quality of learning environments (e.g., educational institutions, teachers, and neighborhoods), and broader socio-economic, cultural and political contexts. Building on this framework, our study focuses specifically on educational inequalities that arise from difference in individual characteristics, particularly gender, cognitive skills, and socio-economic status. Additionally, we extend this analysis by examining the influence of birth month, which, while not explicitly addressed in the OECD framework, has been shown in prior research to play a significant role in shaping educational outcomes^4^.

Family socio-economic status (SES) has received most of the attention in educational inequality research. Meta-analysis has revealed a small to moderate (*r*s = 0.22–0.28) association between SES and academic success across the world^5,6^. In France, socio-economic disparities in pre-academic outcomes are already detectable by age 3, with children from higher-SES families outperforming their peers in early language and early numeracy skills^7^. In addition, although historically among the OECD and European Union countries with the largest gaps between socio-economically advantaged and disadvantaged students, recent trends indicate a relative reduction in these disparities^8^. However, inequalities persist and continue to shape educational trajectories, particularly for students from the most disadvantaged backgrounds. Several factors contribute to these inequalities, including the selective nature of the French education system, which tends to amplify initial disparities rather than mitigate them. For instance, access to high-quality early childhood education and extracurricular activities is often unevenly distributed, favoring children from higher SES backgrounds^9^. Additionally, socio-economic segregation across schools, partly driven by residential zoning policies and selective private schooling, further entrenches these disparities^10^. Longitudinal studies have highlighted that the gap in educational attainment between students from socially advantaged and disadvantaged backgrounds remains relatively stable between the ages of 7 and 11, and increases at an accelerating rate between the ages of 11 and 15^11^. This intensification underscores the importance of understanding and addressing educational inequalities from the earliest years of schooling.

Gender difference in academic achievement have also been extensively studied demonstrating an advantage for girls^12^. However, these differences vary significantly by domain, age, and cultural context. For example, PISA results indicate that this difference vary by domain among 15-year-old students: girls consistently outperform boys in reading comprehension, while boys tend to achieve higher scores in mathematics^8^. Importantly, gender disparities emerge much earlier in development, as girls already show advantages in both early literacy and early numeracy in preschool^13^.These patterns are not universal, however: national differences in educational systems, gender norms, and assessment methods shape the magnitude, and sometimes even the direction of these gaps^14,15^. In France, the gender gap follows the broader OECD trend but with notable nuances. Indeed, gender disparities are already present in preschool, where girls outperform boys in both early language and early numeracy skills at age 3^7^. This early advantage persists into the first years of elementary school, with girls continuing to outperform boys in both language and mathematics^16^.

However, by the middle of first grade, boys outperform girls in mathematics, a difference that persists through secondary school^17^. This gender gap contrasts with patterns in other countries. For instance, in Nordic countries the boy’s advantage in math is smaller^18^. Critically, gender differences also depend on the type of tasks to assess academic performance. Girls typically excel in coursework and sustained, verbal-heavy assessments^19^, whereas boys may perform better in timed, visuospatial tasks^20^ or high-stakes exams^21^. Thus, while gender differences in achievement are robust across cultures, their expression is highly context dependent.

Finally, birth month while receiving less attention, also appears to contribute to educational inequalities. Research suggests that being among the oldest in one’s cohort offers advantages, as older children in the same class often achieve better academic results due to their greater physical, emotional, and cognitive development compared to their younger peers^22,23^. While some researchers suggest that the performance gap between younger and older students diminishes as they progress through school, evidence varies depending on the age range studied. For instance, studies conducted with children aged 5–6 have documented this trend^24^, and more recent work with students aged 6–12 has reported similar patterns, indicating that younger pupils gradually narrow the gap with their older peers^25^. However, in France, this difference persists at age 15, consistently favoring older students^4^, and birth-month disparities are already present in preschool, with older children showing higher early language and early numeracy skills at age 3^7^. Advantage for those born earlier in the calendar year in a given school year could be in part attributed to the amount and duration of parental support prior to kindergarten entry, as older children may benefit from additional time to develop in a home environment before starting school^22^.

Some of these three factors appear to interact with each other in shaping academic achievement. For instance, the impact of birth month is less pronounced for girls compared to boys among fourth and eighth graders^25^. Two possible explanations have been proposed: either girls are less directly affected by their birth month, or they are better able to catch up with their older peers than boys. Similarly, the effect of birth month on academic outcomes is weaker for students from wealthier backgrounds^25^, likely because higher family SES provides access to compensatory resources (e.g., tutoring, enriched learning environments) that mitigate the initial disadvantage associated with younger school entry^26^. A potential interaction between family SES and gender on academic abilities remains unexplored, particularly during early childhood, leaving an important gap in our understanding of how these factors collectively influence educational outcomes.

To better understand how these factors could affect academic achievement, some studies tried to identify the potential mediators of such associations. Most of the studies so far focused on the mediators of the association between family SES and academic abilities with executive functions (EFs) as key mediator of this association. Indeed, a recent meta-analytic structural equation modeling study^27^ synthesized data from 70 empirical articles comprising 58,818 children and confirmed that EFs partially mediate the SES– academic achievement association (indirect effect, *b* = 0.083). The mediation effect was reported in cross-sectional and longitudinal studies, though moderator analyses indicated that the strength of the indirect effect decreases with age. The meta-analysis included diverse populations but emphasized early childhood as a particularly sensitive period for EF development, highlighting its role in reducing SES-based disparities in academic outcomes. Thus, the potential mediators of the association between academic abilities and either gender or birth-month has received little attention to date. In this context, our study aimed to address this gap, to determine whether EFs constitute a mediator of the association not only between academic abilities and family SES but also between academic abilities and gender or birth-month.

EFs are defined as distinct but interrelated higher-order neurocognitive processes, largely dependent on the prefrontal cortex and its interconnected neural networks, that regulate thoughts, behaviors, and emotions to achieve goal-directed outcomes^28,29^. These processes enable individuals to control their behavior and manage cognitive and emotional activities. Three core EFs have been identified: working memory (i.e., the capacity to temporarily hold and manipulate information), inhibitory control (i.e., the ability to control automatisms), and cognitive flexibility (i.e., the capacity to shift attention between tasks or mental sets, adapt strategies in response to changing rules or environmental demands, and flexibly adjust behavior when faced with novel, unexpected, or conflicting information)^30,31^.

EFs emerge during infancy and continue to develop throughout childhood and adolescence^28^. This prolonged developmental trajectory is driven by significant structural changes in the brain’s prefrontal cortex supporting the rapid maturation of these cognitive processes, particularly between the ages of 2 and 5^32^. For instance, EFs undergo rapid and continuous improvements between 3 and 5 years of age with month-by-month changes documented in children’s ability to maintain task rules, inhibit prepotent responses, and flexibly switch between rules in the Dimensional Change Card Sort (DCCS) task due to the functional specialization of the dorsolateral prefrontal cortical networks^31^.

During this critical maturation period, EFs are particularly sensitive to the environment^33^ including family SES^34,35^. In particular, low-SES environments, characterized by increased exposure to stressors such as parental conflict, household instability, and caregiver health difficulties, as well as economic hardships like food insecurity, housing instability, and chronic financial strain^36^, and by limited access to cognitively stimulating materials and activities^37^, negatively affect children’s development.

Gender may contribute to differences in EF development, with some studies reporting earlier maturation in girls, particularly between ages 2 and 5^38,39^. These trends have been linked to behavioral differences, with boys generally displaying more impulsivity and difficulty adapting to novel situations^40^. Such differences are shaped by multiple biological factors, including sex hormones, brain maturation, neural circuitry, and gene–environment interactions,^40,41^. In addition to biological influences, gendered expectations from parents and teachers have been shown to contribute to apparent differences in EF. Adults often hold distinct behavioural expectations for boys and girls, which can shape both opportunities for self-regulation and the way children’s behaviours are interpreted^42^. Parental expectations themselves vary across cultural contexts, and cross-national evidence shows that parents tend to encourage different forms of behavioural regulation in boys and girls, contributing to culturally patterned gender differences in EF^42^. For instance, cross-cultural studies show that teachers tend to rate boys as having more behavioural and attentional difficulties even when direct assessments reveal no gender differences in EF performance^43^. These findings highlight that gendered socialisation and evaluative biases may amplify or produce perceived gender differences in EF. However, gender differences in EF remain modest overall, and within-gender variability often exceeds between-gender variability. Moreover, cultural context and task characteristics can influence observed effects, which are best interpreted as context-sensitive tendencies rather than universal developmental patterns^40,42,44^.

Finally, EFs are recognized as significant predictors of both academic and pre-academic abilities. In school-aged children, longitudinal and cross-sectional studies have shown that EF skills—including working memory, inhibitory control, and cognitive flexibility—are associated with later performance in mathematics, reading comprehension, and general academic achievement^45–47^. In early childhood, EF also predicts pre-academic abilities, including vocabulary, syntactic knowledge, phonological awareness, and early numeracy skills such as counting and number comparison^48–50^. These associations have been demonstrated in children as young as 3 years and across both cross-sectional and longitudinal designs^46,47^. Moreover, longitudinal studies indicate that the direction of this association changes with age. During preschool, EF and early academic skills show bidirectional associations—particularly between EF and mathematics—whereas by kindergarten, EF increasingly emerges as a predictor of later academic performance and not the other way around^51^. Collectively, these findings support the relevance of EF for early learning.

Given that (a) family SES, gender, and birth month affect academic abilities and EFs development from an early age and (b) EFs is a predictor of both pre-academic and academic abilities, we investigated whether EFs mediate the association not only between pre-academic abilities and family SES but also between pre-academic abilities and gender or birth month. To do so, we used a nationally representative dataset of 2,618 children in their first year of formal schooling (age 3 in France). Data were collected on children’s EF abilities (inhibitory control, cognitive flexibility, and working memory), early oral language abilities (syntax, vocabulary, and processing), and early numeracy abilities (numerical thinking and spatial thinking). Using structural equation modeling, we investigated whether (a) family SES, gender and birth month affect pre-academic abilities, (b) the effects of such factors on pre-academic abilities interact with each other and (c) EFs mediate the association between pre-academic abilities and family SES, gender or birth-month. This mediation modeling choice is theoretically and empirically grounded. Theoretically, EF are considered foundational for academic learning, as they support attentional control, instruction-following, and adaptive problem-solving^52^. Empirically, while reciprocal associations between EF and academic skills have been proposed, longitudinal studies more consistently show that early EF predicts later academic performance, whereas reverse pathways are less robust or not systematically observed^53,54^. This asymmetry supports the plausibility of EF as a mediating mechanism through which sociodemographic factors influence early academic outcomes.

## Results

### Family SES computation index

The results of the PCA we performed revealed that the principal first component (PC1) explains 58% of the variance with an eigenvalue of 1.74. Factor loadings are 0.73 for education level, 0.76 for occupation level and 0.79 for household income, indicating that all variables contribute significantly to PC1. We therefore extracted the first component to create a family SES index. We summarized the descriptive statistics and coefficient of correlations in Table 1.

**Table 1:** Summary of correlations, means, standard deviations and percentage of missing data for the study variables

Measures	1	2	3	4	5	6	7	8	9	10	11	12	13	14
**Individual backgrounds**														
1. Birth month	-													
2. Gender (girl=1)	−0.02	—												
3. Education level	−0.01	0.01	—											
4. Occupation level	(0.00)	0.01	0.33	—										
5. Household income	0.01	−0.06	0.37	0.42	—									
6. Family SES index	(0.00)	(−0.02)	0.73	0.77	0.79	—								
**Executive functions**														
7. Working memory	−0.21	0.03	0.08	0.08	0.05	0.09	—							
8. Inhibitory control	−0.27	0.13	0.13	0.07	0.07	0.12	0.30	—						
9. Cognitive flexibility	−0.24	0.10	0.10	0.06	0.07	0.10	0.26	0.55	—					
**Early oral language abilities**														
10. Vocabulary	−0.26	0.10	0.24	0.22	0.19	0.28	0.22	0.32	0.31	—				
11. Syntax	−0.25	0.11	0.17	0.17	0.13	0.21	0.22	0.31	0.29	0.59	—			
12. Processing	−0.22	0.12	0.18	0.19	0.14	0.22	0.14	0.24	0.27	0.56	0.52	—		
**Early numeracy abilities**														
13. Numerical thinking	−0.33	0.13	0.27	0.20	0.26	0.32	0.27	0.36	0.30	0.49	0.43	0.41	—	
14. Spatial thinking	−0.24	0.13	0.23	0.13	0.19	0.24	0.19	0.28	0.21	0.39	0.33	0.29	0.59	—
*N*	2.618	2.618	1.887	2.239	2.306	1.808	2.370	2.425	2.391	2.472	2.472	2.472	2.617	2.617
*M*	—	—	5.96	48.31	—	—	1.32	0.59	0.46	6.74	6.62	8.14	6.56	4.70
*SD*	—	—	2.61	15.07	—	—	1.33	0.28	0.22	2.94	2.88	3.19	3.15	1.55
*Missing %*	0	0	27.89	14.44	11.92	30.94	9.44	7.34	8.64	5.54	5.50	5.50	0	0

For correlations, all coefficients ≥ 0.03 are significant at *p* < 0.001, coefficients below are significant between *p* < 0.05 and *p* < 0.01. Coefficients in parentheses are not significant. For gender, boys and girls are coded 0 and 1 respectively.

### Regression model analysis

Family SES, gender and birth month were all related to the pre-academic abilities (see Table 2). A higher SES index was associated with higher pre-academic abilities scores (*β* = 0.43, *p* < 0.001). Girls compared to boys displayed better pre-academic abilities scores (*β* = 0.17, *p* < 0.001). Finally, the earlier the child was born in the year the better the pre-academic scores (*β* = −0.39, *p* < 0.001). Together, these main effects account for 37% of the variance of the pre-academic scores.

**Table 2:** Summary of principal effects estimates from multiple regression

Predictors	*B*	95%CI	*β*	*p*-value
Family SES index	*0.33*	[0.30, 0.37]	0.43	**<** **0.001**
Gender (girl=1)	0.26	[0.20, 0.32]	0.17	**<** **0.001**
Birth month	−0.09	[−0.10, −0.08]	−0.39	**<** **0.001**

*B* unstandardized coefficients, *95% Cl* 95% confidence interval, *β* = standardized coefficients, *p*-value = significance level. RMSEA = 0.03; CFI = 0.99; R^2^ = 0.37.

When the two-way and three-way interactions were integrated in the model, the main effects of family SES, gender and birth month on pre-academic scores remained significant (all *p*s < 0.001) (see Table 3). Nevertheless, we found no interaction between family SES and gender (*β* = 0.08, *p* = 0.62), family SES and birth month (*β* = 0.05, *p* = 0.75), gender and birth month (*β* = 0.02, *p* = 0.84) or between family SES, gender and birth month (*β* = −0.11, *p* = 0.49) on pre-academic scores.

**Table 3:** Summary of the main and interaction effects estimated from multiple regression

Predictors	*B*	95%CI	*β*	*p*-value
Family SES index	*0.31*	[0.08, 0.54]	0.40	**<** **0.001**
Gender (girl = 1)	0.25	[0.20, 0.32]	0.16	**<** **0.001**
Birth month	−0.09	[−0.10, −0.08]	−0.41	**<** **0.001**
Family SES index*Gender	0.04	[−0.12, 0.19]	0.08	0.62
Family SES index*Birth month	0.01	[−0.03, 0.04]	0.05	0.75
Gender*Birth month	0.002	[−0.02, 0.02]	0.02	0.84
Family SES index *Gender*Birth month	−0.01	[−0.03, 0.02]	−0.11	0.49

*B* unstandardized coefficients, *95% Cl* 95% confidence interval, *β* = standardized coefficients, *p*-value = significance level. RMSEA = 0.03; CFI = 0.99, R^2^ = 0.37.

### Mediation model analysis

**Fig. 1:**
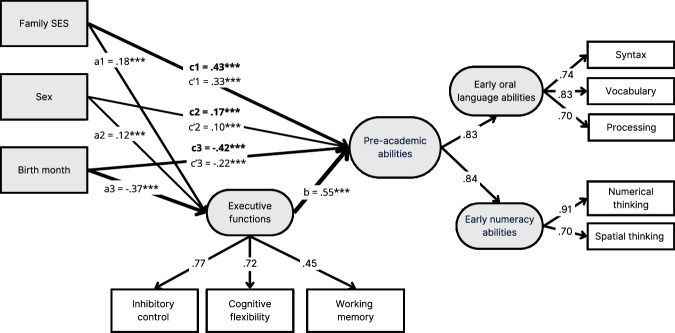
Mediation model. Mediation model of EFs of the relations between family SES and pre-academic abilities, gender and pre-academic abilities and between birth month and pre-academic abilities. Paths a1, a2, a3, b, and c’1, c’2, c’3 report the standardized beta for each corresponding direct effect. Paths c1, c2 and c3 report the total effect. All effects have a significance level of *p* < 0.001. RMSEA = 0.03; CFI = 0.99; R^2^ = 0.61. Girls are coded as 1 and boys as 0. Covariances are not reported in the figure.

Fit indices for the mediation model were very good, *χ2*(35) = 115.97, *p* < 0.001, RMSEA = 0.03, SRMR = 0.02, TLI = .98 and CFI = .99. Family SES (*β* = 0.33, *p* < 0.001), gender (*β* = 0.10, *p* < 0.001) and month birth (*β* = −0.22, *p* < 0.001) have an effect on pre-academic scores as well as on EF scores, with respectively *β* = 0.18, *p* < 0.001 for family SES, *β* = 0.12, *p* < 0.001 for gender and *β* = −0.37, *p* < 0.001 for birth-month. EF scores have also an effect on pre-academic scores, *β* = 0.55, *p* < 0.001. Finally, EF scores mediated the association between pre-academic scores and not only family SES (*β* = 0.10, *p* < 0.001, 23% of the total effect), but also gender (*β* = 0.10, *p* < 0.001, 39% of the total effect) and month of birth (*β* = −0.20, *p* < 0.001, 51% of the total effect). For contrast analysis, the indirect effect of birth month was stronger than that of gender (*β* = 0.27, *p* < 0.001), and the indirect effect of family SES also differed from that of month of birth (*β* = 0.30, *p* < 0.001), indicating a stronger influence of month of birth on pre-academic abilities. However, no significant difference was found between the indirect effects of gender and family SES (*β* = 0.03, *p* = 0.24). All together, these effects explained 61% of the variance of the pre-academic scores. (Fig. 1).

## Discussion

The aim of this study was to better understand early educational inequalities related to family SES, gender and month of birth, using a nationally representative database of 2,618 children aged 3.5 in their first year of formal schooling. Specifically, we investigated whether (a) family SES, gender and birth month affect pre-academic abilities, (b) the effects of such factors on pre-academic abilities interact with each other and (c) EFs mediate the association between pre-academic abilities and family SES, gender or birth-month.

In line with the literature, we report that family SES^5,6^, gender^12^ and month of birth^22,23^ predict pre-academic abilities as early as age 3. Family SES appears to be the most important predictor, followed by month of birth and gender. This may reflect the advantages conferred by access to enriched home learning environments, higher-quality childcare, and increased exposure to cognitively stimulating activities, which are more accessible to families with greater resources^55,56^. Girls perform better than boys, possibly due to early difference in self-regulation, language acquisition, and attentiveness, which have been linked to gender-based developmental trajectories^57,58^. Similarly, being the oldest in one’s age cohort has a significant advantage on pre-academic abilities, likely driven by greater neurological and emotional maturity, as well as more time to develop foundational skills before formal schooling begins^59^. These findings align with the concept of relative age effects, where older children often perform better due to age-related cognitive and physical advantages^23^.

As opposed to what is reported in older children, no interaction effect was found between family SES and month of birth or between birth month and gender^25^. In addition, we detected no interaction between family SES and gender. These interaction effects could arise later, as disparities related to SES, gender, and age compound over time through differential access to resources, opportunities, and social expectations. These mechanisms warrant further investigation in longitudinal studies to better understand their evolving impact on academic trajectories.

These three factors all had an effect on EF abilities. Month of birth appeared to be the strongest predictor at this age, followed by family SES and gender. The effect of month of birth is partly explained by the fact that at the age of 3, children are in a sensitive period for the development of the brain structures that support EFs^32^. Any difference in birth month can therefore produce difference in EF abilities^31^. Family SES affect EF abilities because at this age their development is particularly sensitive to their environment^33^. Factors such as stress, financial and relationship problems within the family home influence the development of EFs^36^. Parenting practices play a role in developing such abilities: children from privileged backgrounds tend to be more exposed to educational activities and stimulating materials that foster EFs^37^. In addition, factors such as stress, financial and relationship problems within the family home influence the development of EFs^36^. Finally, the effect of gender on EF abilities are in line with studies reporting that boys appear to be more impulsive and have more difficulty adapting to new situations than girls^40^. Again, gender specific parental practices might be at the root of such difference with parents being more prone to expect self-regulation from girls than boys^60,61^.

Consistent with the literature, EF abilities influenced pre-academic abilities presumably because EF abilities facilitate the acquisition of information and by helping children to remain focused on the information relevant to the task despite distractions^46,47,62^.

Finally, we found that EFs mediated the effects of family SES, gender and birth month on pre-academic abilities. We thus extended previous finding on the mediating role of EFs on the association between Family SES and pre-academic abilities^27^ to two additional factors supporting educational inequalities. Importantly, the mediation effects of EFs on the association between pre-academic abilities and birth month or gender are greater than the one for family SES. The mediation effect of EFs could be more pronounced for birth month because early childhood is a pivotal developmental period for EFs with sharp improvement of such abilities^28,31^ supported by the rapid maturation of prefrontal brain regions.

Overall, this study highlights that educational disparities linked to family SES, gender, and birth month are already detectable during early preschool. While these demographic characteristics cannot be changed, our findings underscore the importance of EFs as a common, malleable mechanism underlying these early inequalities^63,64^. This suggests that promoting EF development from an early age, through enriched structured play or targeted support^65^, may represent a promising pathway for reducing disparities rooted not only in socioeconomic disadvantage, but also in normative age differences and gender-based challenges in self-regulation^64^. Future research should examine the effectiveness of such interventions in mitigating multiple forms of early academic disadvantage.

The current study has several limitations. Firstly, this is a cross-sectional study, which limits our ability to draw causal conclusions. While our mediation modeling choice is theoretically and empirically grounded, EF are considered foundational for academic learning^52^, and longitudinal studies more consistently show that early EF predict later academic performance than the reverse^53,54^, we acknowledge that the directionality of associations between sociodemographic factors, EF, and pre-academic abilities cannot be firmly established from cross-sectional data alone. This design restricts inferences about developmental processes and the temporal unfolding of mediation effects. Therefore, future longitudinal studies are essential to clarify the sequencing of these associations, track changes over time, and strengthen the causal interpretation of EF as a mechanism linking early inequalities to school readiness. Secondly, our analytic strategy focused on broad latent constructs, aggregating multiple EF dimensions (inhibitory control, cognitive flexibility, and working memory) and various pre-academic skills (oral language and numeracy). While this approach enhances reliability and reflects shared variance across indicators, it does not allow identification of the most influential subcomponents of EFs for each sub-domains of pre-academic skills. Future research should employ more fine-grained models capable of disentangling the specific contributions of individual EF dimensions and academic skills. This is critical to develop effective educational or clinical interventions relying on fostering the most influential EF for the sub-domain of pre-academic skills of interest. Thirdly, this study only considered EFs as a potential mediator of the effects of family SES, gender, and birth month on pre-academic abilities. However, other factors such as metacognition could mediate such association^66^. Finally, our study focused exclusively on “cool” executive functions, assessed in emotionally neutral contexts. However, “hot” EF, such as self-control and delay of gratification, which are recruited in emotionally salient or motivationally charged situations, have also been linked to academic outcomes^67^. Future research should incorporate both hot and cool EF measures to provide a more comprehensive understanding of the self-regulatory mechanisms underlying early educational inequalities.

In conclusion, we provided the first evidence that educational inequalities related to gender and birth month can be explained in part by the effect of EFs, as originally reported for family SES, from age 3 at the start of formal schooling in France. They speak to the importance of considering these factors to better understand the emergence of educational inequalities and to design intervention to mitigate such effects from the start of schooling.

## Methods

### Participants

We used a sample of 2,618 preschoolers (mean age in months ± SD: 42.61 ± 3.41, girls/boys *n* = 1254/1364) constituted by the Direction de l’Évaluation, de la Prospective et de la Performance (DEPP) of the French Ministry of Education in 2021. The sample was drawn using a stratified random procedure by the Ministry itself to ensure representativeness at the national level. As such, there were no specific inclusion or exclusion criteria applied by the research team. The sample encompasses a diverse range of geographical areas (urban, suburban, and rural) and school sectors (public, private, and public priority education areas). This diversity ensures a broad spectrum of family socio-economic backgrounds (see Table 4). The distribution of participants across birth months was relatively balanced (ranging from 7% to 9.2% per month), and a chi-square goodness-of-fit test indicated no significant deviation from uniformity, *χ*^2^(11) = 16.42, *p* = 0.13. The study was approved by the National Council for Statistical Information (CNIS) (visa n° 2021A091ED). Regarding ethical procedures, the Ministry of Education is legally authorized to conduct such evaluations within the public education system, under national legislation governing educational research and data protection. All data were collected, anonymized, and processed by the Ministry’s statistical service in compliance with French regulations and the General Data Protection Regulation (GDPR). Both parents and children were informed of the assessment procedures, and participation was not mandatory, families had the right to decline participation if they wished. The study was conducted in accordance with the ethical standards laid down in the 1964 Declaration of Helsinki and its later amendments.

**Table 4:** Sample characteristics compared to the general population

	Sample		General population	
	*N* = 2618		*N* = 717,891	
	*N*	%	*N*	%
**Sex**				
Girl	1254	48.00	351,778	49.00
Boy	1364	52.00	366,113	51.00
**School district**				
Private	261	10.00	83,644	11.70
Public and REP	2148	82.00	584,783	81.50
REP+	209	8.00	49,464	6.90
**Geographical area**				
Urban	2121	81.00	130,302	81.90
Rural	496	19.00	130,302	18.10

### Materials and procedure

The children completed the oral language task and the EFs tasks on a tablet, under the supervision of a trained experimenter, in January 2022. For the numeracy task, the various problem situations were administered directly by the teachers. Both the trained experimenters and classroom teachers followed standardized protocols established by the Ministry of Education and received prior training to ensure consistency in administration across settings. All assessments were conducted within the school setting. Legal guardians completed a questionnaire to provide socio-demographic information.

To assess inhibitory control and cognitive flexibility, an adapted version of the Hearts and Flowers task^68^ was used. In this task, children are shown an image of either a heart or a flower on one side of the screen and are instructed to press the button on the same side when a heart appears (congruent trial) and on the opposite side when a flower appears (incongruent trial). A fixation cross is displayed for 300 ms at the center of the screen before each trial to focus the child’s attention. Each stimulus is shown for 2500 ms, with a 750 ms interstimulus interval. The task begins with a training phase to familiarize the children with the procedure, followed by three blocks of trials: a congruent block with 12 congruent trials, an incongruent block with 12 incongruent trials, and a mixed block comprising 12 congruent and 12 incongruent trials. Accuracy rates were calculated for each block^69^, and scores were computed only for children who completed at least 75% of the trials within each block. Accuracy scores for both the inhibitory control and cognitive flexibility blocks were calculated as the proportion of correct responses across trials, yielding values ranging from 0 to 1. Higher accuracy scores thus indicate better EF performance. Inhibitory control was measured using the accuracy rate from the incongruent block (*α* = 0.81), while cognitive flexibility was assessed using the accuracy rate from the mixed block (*α* = 0.83).

Visuo-spatial working memory was assessed using the Corsi blocks task^70^. In this task, children are shown a sequence of highlighted blocks on the screen and must reproduce the sequence by tapping the corresponding blocks. The sequence increases by one block every two trials. The visuo-spatial memory span is determined by the length of the last correctly reproduced sequence, ranging from 0 to 7.

A French adaptation of the Rapid Interactive Language Test^41^ was used to evaluate three language components: vocabulary, syntax, and the process of learning new words. The vocabulary subtest assesses children’s knowledge of two types of words: those representing semantically meaningful concepts (Nouns with 3 items, Verbs with 5 items) and those aiding in grammatical functions necessary for sentence construction and meaning modulation (Prepositions with 4 items, Conjunctions with 2 items) (see Table 5). The syntax subtest examines children’s understanding of various syntactic structures, including sentences with multiple modifiers like prepositional phrases (3 items), sentences with embedded clauses (3 items), interrogative sentence structures (5 items), and sentences that describe past events (4 items) (see Table 5). The new words learning process evaluates children’s ability to acquire new vocabulary and generalize syntactic structures using those words, assessed through 4 subtests: noun learning (10 items), adjective learning (10 items), verb learning (4 items), and conversion from active to passive voice (2 items) (see Table 5). The assessment begins with three training items designed to familiarize the child with the test procedures. Following this, an audio file plays the question for each item, and the child responds by selecting one of the possible answers displayed on the screen. One point is awarded for each correct answer, except in the noun learning and adjective learning subtests, where two correct responses are required to count as one point (i.e., fast mapping and extension tasks). The total number of correct answers per dimension is then calculated, resulting in three sub-scores.

**Table 5:** Example of item instruction for each subtest associated with the two abilities (Early oral language and early numeracy)

Ability	Subtest	Example of item instructions	Omega (ω)
Early oral language	Vocabulary^a^	“Show me the fireworks.” with four possible image responses: a window, a felt pen, a matchbox, a firework (target)	0.72
Early oral language	Syntax^a^	“Show me the teddy bear on top of the present.” with four possible image answers: a teddy bear under the present, a teddy bear on top of the present (target)	0.70
Early oral language	New words learning process^a^	“The torba is blue! Show me the blue torba.” with four possible image answers: an unknown yellow object, a blue book, a blue train, a blue torba (target)	0.62
Early numeracy	Numerical thinking^b^	“Give me 3 pearls.”	0.88
Early numeracy	Spatial thinking^b^	“Take these two objects and give me the larger one.”	0.80

^a^French adaptation of the rapid interactive language test (Golinkoff et al.^41^; Levine et al., 2020).

^b^French adaptation of the Longitudinally Adaptive Assessment and Instruction (Raudenbush et al.^71^).

A French adaptation of the Longitudinally Adaptive Assessment and Instruction^71^ was used to assess two early numeracy skills: numerical thinking and spatial thinking. The numerical thinking score, comprising 11 items, evaluates the ability to construct numbers to express quantities, stabilize knowledge of small numbers, and use numbers to indicate rank (see Table 5). The spatial thinking score, consisting of 6 items, measures knowledge of shapes and their properties, the ability to mentally manipulate objects in space, and the capacity to build larger structures from smaller components (see Table 5). Each item includes up to three trials. For items with only one trial, one point is awarded for a correct response, while for items with three trials, all three must be answered correctly for the item to be scored as correct.

Legal guardians provided information on their highest level of education, then converting into years of schooling (ranging from 0 to 11, with 11 representing a graduate degree). We used the highest education level of either parent as the indicator for parental education.

Each legal guardian reported their occupation in open-ended format, along with additional details necessary for classification (e.g., number of supervised employees). The coding into the Professions et Catégories Socioprofessionnelles (PCS 2020) system, developed by the Institut national de la statistique et des études économiques (INSEE) to describe French society, was subsequently performed by the DEPP based on the information provided in the questionnaire. Based on this system, four occupational categories were defined and assigned numerical values: highly advantaged (company directors with ten or more employees, executives, higher intellectual professions, and teachers), advantaged (intermediate occupations and retired executives), average (farmers, craftsmen, shopkeepers, and corresponding retirees, employees), and disadvantaged (blue-collar workers, retired blue-collar workers and employees, inactive individuals, and unemployed individuals who have never worked). We used the highest occupational category of either legal guardian to determine the occupation level.

Parents reported their total monthly income for a standard month before deducting taxes for the whole household. A total of fifteen answers were possible, from less than 400 euros to more than 10,000 euros. We used the selected category as an indicator of the household’s average income.

### Statistical analysis

All analyses were performed using R-statistical software, version 3.6.3^72^.

We created an index score for the family SES by running a principal component analysis (PCA) including the three variables collected, namely education level, occupation level and household income^73^. This index was created by extracting the first component. Although these indicators differed in measurement scale, education level was converted into years of schooling (ranging from 0 to 11), occupation level was coded into four ordered categories based on the French PCS 2020 classification (from “disadvantaged” to “highly advantaged”), and income was reported using fifteen ordered categories ranging from less than 400€ to more than 10,000€, methodological research supports the use of PCA with such ordinal or mixed data under certain conditions, especially when the variables are conceptually aligned and moderately intercorrelated^74,75^.

For the gender variable, girls were coded as 1 and boys as 0. For the birth month variable, months were coded numerically, with January as 1 and December as 12. Table 1 provides the descriptive statistics and correlation coefficients between all variables considered.

We conducted all multiple regression and mediation analysis using structural equation modeling with the *lavaan* package^76^. Latent variables were created to represent EFs, early oral language abilities, and early numeracy abilities. Additionally, a second-order latent variable for pre-academic abilities was constructed, combining early oral language and early numeracy abilities to capture their shared contribution. We first conducted a multiple regression model to examine the main effects of the family SES, gender, and birth month on pre-academic abilities. We ran another regression model to detect potential interaction effects between these three variables on the pre-academic abilities. Finally, in the mediation model we assessed whether a general EF ability mediated the association between pre-academic abilities and each of the three predictors (i.e., family SES, gender, month of birth). To examine whether the mediation effects significantly differed from one another, contrasts between the indirect effects were defined and tested^77^. We analyzed the pattern of missing data using the MICE package^78^, with missingness rates below 15% for all variables except education level and family SES index (respectively 28% and 31% of missing values)^79^. As the data were missing at random (MAR), we used full information maximum likelihood (FIML) estimation to handle missing data in the SEM^80^. We applied robust maximum likelihood estimation^81^ to address non-normality. Model fit was evaluated using the following criteria: Tucker-Lewis Index (TLI) and Comparative Fit Index (CFI) values ≥ 0.95, and Standardized Root Mean Square Residual (SRMR) and Root Mean Square Error of Approximation (RMSEA) values ≤ 0.05^82^. We used standardized estimates to control for scale difference and compare the relative impact of predictors. Finally, for the mediation model we applied a Bonferroni correction, setting the significance threshold at α = 0.007 (0.05 divided by the 7 tests in the SEM).

Prior to estimating the structural paths, we conducted confirmatory factor analyses (CFA) to validate the measurement models for the latent constructs. For EFs, a one-factor model including inhibitory control, cognitive flexibility, and working memory showed excellent fit to the data (CFI = 1.000, TLI = 1.000, RMSEA = 0.000, SRMR = 0.000). All factor loadings were statistically significant and exceeded conventional thresholds, with standardized loadings ranging from 0.40 to 0.78 (all *p*s < 0.001). For academic abilities, a second-order CFA was estimated with two first-order latent variables—early oral language abilities (syntax, vocabulary, processing) and early numeracy abilities (numerical thinking, spatial thinking)—and a second-order latent factor pre-academic abilities. This modelling strategy was selected because early numeracy abilities were assessed with only two indicators. As a latent factor defined by two observed variables is only weakly identified and requires additional constraints that were not theoretically justified, estimating two separate latent variables of equal psychometric robustness was not feasible. The second-order structure therefore provided a theoretically coherent and statistically appropriate solution, allowing us to integrate both domains within a unified construct while maintaining their conceptual distinction at the first-order level. This model demonstrated excellent fit (CFI = 0.996, TLI = 0.990, RMSEA = 0.041, SRMR = 0.014). All factor loadings were significant (*p* < 0.001), with standardized estimates ranging from 0.70 to 0.91. These results support the validity of the measurement model for academic abilities.

## Data Availability

The data supporting the findings of this study are derived from administrative databases of the French Ministry of National Education. Access to these data is restricted due to legal and ethical constraints, but may be granted upon reasonable request and subject to authorization by the Ministry.

## Code availability

Code is available from the first author.
